# Consistency and applicability of different brief screen instrument of cognitive function in elderly population

**DOI:** 10.1186/s12883-021-02048-4

**Published:** 2021-03-01

**Authors:** Lixia Lu, Lin Chen, Weiwen Wu, Yang Wang, Zhenbao Liu, Jun Xu, Qianhong Yang, Jun Zhao, Liangxian Liu, Hui Yu

**Affiliations:** Department of Neurology, Qingpu Branch of Zhongshan Hospital, Fudan University, 1158 East of Park Road, Qingpu District, Shanghai, 201700 China

**Keywords:** Cognitive assessment screening instrument, Consistency, Applicability, Cognitive dysfunction, Mini-mental state examination

## Abstract

**Background:**

Screening for cognitive impairment (CI) is often hampered by lack of consensus as to which screening instrument to use. The aim is to assess the consistence and applicability of different CI screening tools.

**Method:**

In a cross-sectional study from October 2017 to September 2018 in 7 communities in Shanghai, China, elder (≧60) residential volunteers with no history of major cardiovascular diseases, cancers and other comorbidities known to affect cognitive functions were recruited. The participants underwent tests with 7 cognitive function screening instruments. Multivariate linear regressions were performed to test correlations between demographic characteristics, including gender, age, education, and marital status, with cognitive test scores. Mini-Mental State Examination (MMSE) score adjusted according to the correlation coefficients was used to detect CI with a cutoff of 24. Other cognitive function scores were compared between participants with and without CI. In addition, Pearson’s correlation test was used to detect association between different test scores.

**Results:**

172 participants with relatively low education levels were included. Age and education showed significant association with cognitive test scores. Using adjusted MMSE, 39.6% of participants were identified with CI, while the percentage was 87.2% when adjusted Montreal Cognitive Assessment (MoCA) with cutoff of 26 was used. Analysis of “abnormal” test scores showed that MMSE had the highest percentage of valid data (98.8%). MoCA and Isaacs test of Verbal Fluency (VF) score had correlation with most the other scores, while MMSE only significantly associated with VF and MoCA.

**Conclusions:**

MMSE may still present the most applicable tools for quick screen of cognitive functions, especially when environmental conditions may interfere with participants’ attention.

## Background

Dementia affect may lead to compromised capability of independent living, causing health concerns as well as great economic burden in society. Aging was the most important risk factor of dementia and senile cognitive impairment has emerged as one of the major public health challenges [1, 2]. Cognitive impairment (CI) is closely associated with higher risk of developing dementia [3]. Early recognition of cognitive problems may provide opportunities to address possible reversible causes, or refer for further evaluation [4].

Although the importance of CI has been well recognized, rarely an accurate estimation of CI prevalence is available in a clinical study. It has been reported that a large proportion of older adults with dementia remain undiagnosed [5]. For example, China has the largest population of patients with dementia, however there lacks accurate estimation of prevalence and incidence, impeding the effective care [6]. The applicability and accuracy of instruments for screening and diagnosis of CI may be an important cause. Routine screening for cognitive impairment is often hampered by time constraints, poor knowledge of screening instruments and lack of consensus as to which screening tool is best.

Detailed clinical diagnosis of CI is a complex process [7]. In practical situations, cognitive testing is more often accomplished using brief cognitive screens such as MMSE or MoCA [8–10]. Many screening tools have been developed, often with overlapped cognitive domains and different methods to assess them [11]. There lacks consensus on which cognitive assessment instrument should be used in various clinical conditions [12–14]. In addition, it is very common that older adults with dementia have high percentage of illiteracy and low educational background, which affect the effectiveness of cognitive screening tools [15]. Moreover, few cognitive assessment tools have been validated in Asia [16].

It is of great importance that a practical and accurate instrument is used in assessing cognitive functions, especially in a short screening process when dedicated evaluation are often impossible. There have rarely been studies concerning the applicability of different cognitive assessment tools in Asia, especially in Chinese population. Here, we present a cross-sectional study assessing cognitive functions using 7 different tools and discuss the practical value of these instruments.

## Methods

### Study design and subjects

The original study was a cross-sectional survey in community setting aiming to assess the prevalence, risk factors and cerebral small vessel imaging characteristics of elder population with cognitive impairment. From October 2017 to September 2018, participants were recruited through on-site visit of 7 communities in Shanghai, China, including:

- Jinze Community, No. 271, Nurture Road, Jinze Town, Qingpu District, Shanghai, China;
- Xujing Community: No. 1088, Xumin Road, Xujing Town, Qingpu District, Shanghai, China
- Xianghuaqiao Community: No.1, Lane 1195, Xinqiao Road, Qingpu District, Shanghai, China
- Daying Community, Xianghuaqiao Street, Qingpu District, Shanghai, China;
- Huaxin Community: 800 Huazhi Road, Huaxin Town, Qingpu District,Shanghai, China;
- Liantang Community: No. 3619, Zhufeng Road, Liantang Town, Qingpu District, Shanghai, China;
- Shenxiang Community: No 39 Quchi Road, Zhujiajiao Town, Qingpu District, Shanghai, China

Each community were with a population from 40,000 to 200,000. Elder (≧60) residents were invited through home visit to participate in the study. Briefly, flyers and other forms of notices were distributed with the help of neighborhood committees in each community in a period of 2 weeks before an on-site public free health consulting for elder people was given in each community. The attendants of all the event were registered and then 300 participants who met the age requirement and were without reported significant diseases were randomly selected and further recruited by a door-to-door visit.

The participants were considered eligible if they have a permanent resident status in Qingpu district, age ≥ 60 years, volunteered to join the study and sign the informed consent. The excluded individuals were those with a history of stroke or diagnosed dementia; history of brain-related diseases such as brain tumors and hydrocephalus; small blood vessel related white matter lesions such as multiple sclerosis; MR contraindications such as cardiac implant pacemakers, implantable defibrillators, cardiac stents, cochlear and other metal implants, claustrophobia; those who were unable to cooperate independently to complete the study; and other serious physical and mental illnesses that may lead to loss of follow-up.

The participants underwent examinations including brain MRI, tests of blood and urine samples, as well as various cognitive functional tests. All participant signed informed consent form and the study was approved by the Ethical Committee of Zhongshan Hospital affiliated to Fudan University, Qingpu Branch.

### Cognitive functional tests

#### MMSE

Mini-Mental State Examination (MMSE) [17] is one of the most common cognitive function tests used in various situations [18]. The test contains 30 items that covers various domains (instant memory, delayed recall, attention and computational power, orientation, object naming, reading comprehension, speech comprehension, language retelling, speech expression, and visual space ability). A cutoff of ≤24 points was usually used as recognition of cognitive impairment [18, 19]. The validity and reliability of Chinese version of MMSE has been verified previously [20, 21].

#### MoCA

The Montreal Cognitive Assessment (MoCA) [22] was developed to screen mild cognitive impairment. The test added assessments in 7 domains (visuospatial/executive function, naming, memory, attention, language, abstraction and delayed recall) to a total of 30 points. MoCA has been validated and widely used in cognitive assessment of Chinese population [23, 24].

#### SDMT

The Symbol Digits Modalities Test (SDMT) [25] is a commonly used screening instrument for neurological dysfunction. Participants are required to match nine abstract symbols paired with numerical digits. The applicability of SDMT in Chinese population has also been tested previously [26].

#### SCWT

The Stroop Color and Word Test (SCWT) [27] is a neuropsychological test designed to measure the Stroop Effect, which usually occurs due to interference when processing two simultaneous stimuli, and is particularly sensitive to a reduced speed of information processing [28]. During the test, participants will be asked to name of the color given in black wording (W) and then in color block (C), then CW card featured the names of colors in incongruent color words was used. The tests is scored based on the completion times and correct numbers [29]. SCWT has also been adopted in various studies of cognitive functions in Chinese population [30, 31].

#### DS

The Wechsler Adult Intelligence Scale-Digit Spans Test (DS) [32] mainly test memory storage capabilities and numerical skills associated with the operational verbal memory function. During test, single digit numbers are read to the participant at a rate of one per second then asked to be repeated orally. There were two subtests of tests: In DS Forward test, participant will be asked to recall a series of random single digits, varies in length from three to nine, according to the reading order. While in DS Backward test, the random single digits with length varies from two to eight, need to be recalled in reverse order. The Forward and Backward tests measure short-term memory capacity and working memory capacity, respectively. It has been proposed that DS is related to general intelligence [33]. DS has also been used to assess cognitive functions in in Chinese subjects [34].

#### TMT

The trail-making test (TMT) [35] is designed to measure processing functions including scanning, visual attention, mental flexibility, executive functions, and processing speed [36–38]. In TMTA test, participants will be asked to connect 25 consecutive numbers, while in TMT-B, mixed numbers and letters need to be connected in required order. The test is scored based on the completion times and errors. Diagnostic accuracy of TMT for cognitive impairment in Chinese has been suggested [39] and widely applied in various studies [40, 41].

#### VF

In the Isaacs test of Verbal Fluency (VF) [42], participants are asked to provide as many words within a specific category as possible in 60 s. Verbal fluency test is a simple tool to assess neurological functions and has also been applied in cognitive functions in Chinese studies [43].

The above tests were performed to score the cognitive functions of the participants, the tests and score methods of different tests are listed in Table 1. All the tests used in the current study have been tested in Chinese population as shown in the above references.

**Table 1:** Tests and scoring systems used in this study

Tests	Score	Note
MMSE	MMSE score	
	AdjustMMSE	Age and Education ajusted MMSE score
SDMT	Numbers	Correct number of substitutions in 90 s
	Missing	Number of remindings to the tested subject that a blank was missed
SCWT	W	Correct numbers of reading names of colors printed in black ink (total 50)
	WT	Time to finish W
	C	Correct numbers of naming color patches (total 50)
	CT	Time to finish C
	CW	Correct numbers of naming color of a printed word with dismatched corlor and word (total 50)
	CWT	Time to finish CW
	LIS	W/C
	HIS	CW/C
	TI	CWT - [(WT + CT)/2]
	EI	(50-CW) - [(WE + CE)/2]
	IS	CWT+[(50-CW)x2x(CWT/CW)]
DS	Forward	
	Backward	
	Total	
TMT	A-E	Errors in TMT-A table
	A-T	Time to finish a TMT-A table
	B-E	Errors in TMT-B table
	B-E	Time to finish a TMT-B table
	Errors	Sum of errors in both table
	Time	Time to finish both table
VF	Animal-15 s	Number of animals named in 0–15 s
	Animal-30s	Number of animals named in 15–30 s
	Animal-45 s	Number of animals named in 30–45 s
	Animal-60s	Number of animals named in 45–60 s
	Animals	Total number of animals named in 90 s
	Objects-15 s	Number of Objects (piece of furniture) named in 0–15 s
	Objects -30s	Number of Objects (piece of furniture) named in 15–30 s
	Objects -45 s	Number of Objects (piece of furniture) named in 30–45 s
	Objects -60s	Number of Objects (piece of furniture) named in 45–60 s
	Objects	Total number of Objects (piece of furniture) named in 90 s
	Alternated -15 s	Number of Alternated (animal and object) named in 0–15 s
	Alternated -30s	Number of Alternated (animal and object) named in 15–30 s
	Alternated -45 s	Number of Alternated (animal and object) named in 30–45 s
	Alternated -60s	Number of Alternated (animal and object) named in 45–60 s
	Alternated	Total number of Alternated (animal and object) named in 90 s
	VF score	Total number of objects named in 3 tests
MoCA	Visuospatial/Executive	
	Naming	
	Memory	
	Attention	
	Language	
	Abstraction	
	Delayed Recall	
	MOCA	Total score of upper 7 categories
	AdjustMoCA	Age and Education adjusted MoCA score

### Data collection

Demographic data of participant that may interfere with cognitive functions, including gender, age, education, and marital status were collected using questionnaire containing basic demographic questions.

Cognitive tests of recruited participants were performed in a study center. Nurses and students from medical school who were trained to use the tools performed the tests and recorded scores as investigators. The investigators were instructed to acquire as complete the test results as possible even when participants could not finish the previous parts of tests.

The medical conditions of participants including current comorbidity and medications were also collected, as well as physical examinations and laboratory tests in the survey process, however these data were not included in the current study.

### Statistical analyses

Statistical analyses were performed using the Windows SPSS software package (version 20.0, IBM Corporation, Armonk, NY, USA). Continuous variables were expressed as mean ± SD, and student t-test was used to test difference between groups. Categorical data was expressed as frequency and percentage, with Chi-square tests used to compare between groups. The Pearson’s correlation test was used to detect association between different variables. To determine the correlated factors of cognitive test scores, multivariate linear were performed. A probability value of *p* <  0.05 was considered statistically significant.

## Results

Total 172 participants were included. As shown in Table 2. The participant population were with an average age of 67.8 years and relatively balanced gender ratio. The education levels of the participant were relatively low with the majority had less than middle school level education (8–9 years). Only 8.8% participants had a high school (11–12 years) or higher level of education.

**Table 2:** Cognitive function related demographic characteristics of study population

		Total (*n* = 172)	Normal (*n* = 104)	Impaired *n* = 66	*P* value
Gender	Male	73 (42.4%)	54	17	0.001
	Female	99 (57.6%)	50	49	
Age		67.8 ± 4.9	67.9 ± 5.0	67.7 ± 4.8	0.844
Marital status	Married	155 (90.1%)	97	56	0.325
	Divorced	2 (1.2%)	2	0	
	Widowed	13 (7.6%)	5	8	
	Missing	2 (1.2%)	0	2	
Education	College (Level 5)	2 (1.2%)	2	0	< 0.001
	High school (Level 4)	13 (7.6%)	10	3	
	Middle school (Level 3)	42 (24.4%)	34	7	
	Elementary (Level 2)	78 (45.3%)	48	29	
	None (Level 1)	31 (21.5%)	10	27	

It is well recognized that age, gender and education level had significant influence on cognitive functions and it is often necessary to adjust test scores to these factors to correctly evaluate CI. Therefore, we adopted an adjustment for CI assessment. Multivariate linear regressions were performed to determine the correlations between demographic characteristics with scores of tests. As shown in Table 3, age and education showed significant association with almost all cognitive test scores, while gender and marital status only associated with a number of scores.

**Table 3:** Correlations between test results and demographic characteristics

		Gender		Age		Education		Marriage	
		Correlation coefficient	*P* value	Correlation coefficient	*P* value	Correlation coefficient	*P* value	Correlation coefficient	*P* value
MMSE		−0.087	0.255	−0.215	0.004	−0.315	< 0.001	− 0.175	0.034
MoCA		− 0.105	0.121	− 0.194	0.003	− 0.528	< 0.001	− 0.113	0.119
SCWT	IS	−0.261	0.003	0.121	0.177	0.13	0.119	0.08	0.41
	TI	−0.233	0.012	0.034	0.716	0	0.996	0.03	0.766
	EI	−0.049	0.601	0.142	0.134	0.156	0.079	0.047	0.647
DST		−0.134	0.074	−0.166	0.023	−0.375	< 0.001	− 0.168	0.038
TMT	Errors	0.113	0.146	0.32	< 0.001	0.285	< 0.001	0.031	0.708
	Time	0.115	0.143	0.141	0.07	0.377	< 0.001	0.013	0.877
VF	Animals	−0.154	0.052	−0.247	0.001	−0.196	0.011	−0.069	0.415
	Objects	−0.031	0.692	−0.251	0.001	−0.258	0.001	−0.11	0.19
	Alternated	−0.109	0.156	−0.377	< 0.001	−0.174	0.02	−0.088	0.282
	Total	−0.097	0.21	−0.31	< 0.001	−0.236	0.002	−0.1	0.226

Then, scores of MMSE and MoCA, the two most commonly tools for CI assessment, were adjusted according to the correlation coefficients found in regressions, with formula:

Adjusted MMSE = MMSE-[0.215x(70-Age)]-[0.315x(Education-4)].

Adjusted MoCA = MoCA-[0.194x(70-Age)]-[0.528x(Education-4)].

After adjustment, CI was determined using either adjusted MMSE with a cutoff of 24 (< 24 considered cognitive function impairment), or adjusted MoCA with a cutoff of 26 (< 26 considered cognitive function impairment) (Table 4). Using adjusted MMSE, 39.6% of participants were identified as with CI. The percentage of CI was 87.2% when adjusted MoCA was used to assess, which is extremely high.

**Table 4:** Number and percentages of the study subjects with effective test results

		Frequency	Percentage	Valid sample percentage
Adjusted MMSE	Normal	104	60.5%	98.8%
	Impair	66	38.4%	
	Missing	2	1.2%	
Adjusted MoCA	Normal	22	12.8%	81.4%
	Impair	118	68.6%	
	Missing	32	18.6%	
SDMT		162		94.2%
SCWT		128		74.4%
DST		168		97.7%
TMT		159		92.4%
VF		170		98.8%

By looking further into the test score, it has been noted that many participants had very low scores in MoCA tests. We reasoned that these results more likely reflected an incomplete test data rather than indicating high prevalence of CI. When the participants with a seemly abnormally low score (≦14) were categorized into “missing” (indicating invalid test data, Table 4), the MMSE identified 38.4% of participants with CI while MoCA identified a 68.6% of CI. We further re-checked on other test scores and categorized those with abnormal data (mainly a missing section in multi-section test, or seemly impossible data such as a 2 s in TMT-B time) as invalid. The results showed that MMSE had the highest percentage of valid data (98.8%), MoCA had only 81.4% valid data. The lowest percentage of valid data was for SCWT (74.4%).

Correlations among different test scores were then tested using linear regression. As shown in Table 5, MoCA and VF score had correlation with most the other scores, while MMSE only significantly associated with VF and MoCA.

**Table 5:** Correlations among different cognitive functional tests

		mmse	SCWT TI	SCWT EI	SCWT IS	TMTErrors	TMTtime	Vftotal	MOCA
mmse	Coefficient	1	−0.04	−0.066	−0.132	− 0.152	−0.086	0.527	0.576
	*P* value		0.655	0.458	0.131	0.054	0.277	< 0.001	< 0.001
SCWT TI	Coefficient	−0.04	1	0.166	0.779	−0.017	0.027	−0.143	− 0.063
	*P* value	0.655		0.059	0	0.847	0.759	0.103	0.474
SCWT EI	Coefficient	−0.066	0.166	1	0.586	0.09	0.109	−0.158	−0.205
	*P* value	0.458	0.059		0	0.308	0.216	0.071	0.019
SCWT IS	Coefficient	−0.132	0.779	0.586	1	0.105	0.057	−0.254	−0.157
	*P* value	0.131	0	0		0.231	0.515	0.003	0.069
TMTErrors	Coefficient	−0.152	−0.017	0.09	0.105	1	0.567	−0.187	−0.32
	*P* value	0.054	0.847	0.308	0.231		0	0.017	< 0.001
TMTtime	Coefficient	−0.086	0.027	0.109	0.057	0.567	1	0.022	−0.265
	*P* value	0.277	0.759	0.216	0.515	0		0.785	0.001
Vftotal	Coefficient	0.527	−0.143	−0.158	− 0.254	−0.187	0.022	1	0.514
	*P* value	< 0.001	0.103	0.071	0.003	0.017	0.785		< 0.001
MOCA	Coefficient	0.576	−0.063	−0.205	− 0.157	− 0.32	− 0.265	0.514	1
	*P* value	< 0.001	0.474	0.019	0.069	0	0.001	< 0.001	

Since MMSE had the most complete data, and the CI recognized with adjusted MMSE appeared to fall in a reasonable percentage, we then compared the test scores between participants with normal and impaired cognitive functions according to the adjusted MMSE score. The results (Table 6) showed that except for SCWT, all other test scores were significantly different between the two groups, indicating a difference in cognitive function between the groups could be detected with most the test tools.

**Table 6:** Correlations between other functional tests with MMSE cognitive impairment diagnosis

		Normal	Impaired	*P* Value
SDMT	Correct *n* = 162	20.1 ± 11.8	6.8 ± 0.9	< 0.001
	Missing *n* = 157	0.36 ± 0.63	0.52 ± 1.19	0.292
SCWT	LIS *n* = 128	1.02 ± 0.11	0.98 ± 0.21	0.17
	HIS *n* = 128	0.95 ± 0.20	0.94 ± 0.24	0.863
	TI *n* = 129	43.9 ± 26.0	43.5 ± 36.2	0.946
	EI *n* = 129	3.02 ± 9.14	2.48 ± 12.1	0.79
	IS *n* = 132	99.1 ± 54.9	106.8 ± 63.9	0.502
DST	Forward *n* = 172	7.34 ± 1.64	5.66 ± 1.61	< 0.001
	Bacward *n* = 168	3.58 ± 1.12	2.58 ± 1.12	< 0.001
	Total *n* = 168	10.91 ± 2.27	8.25 ± 2.04	< 0.001
TMT	A-Errors *n* = 167	18.3 ± 11.4	22.8 ± 9.8	0.009
	A-Time *n* = 162	78.7 ± 32.2	76.9 ± 47.0	0.774
	B-Errors *n* = 161	33.3 ± 27.5	43.8 ± 26.7	0.02
	B-Time *n* = 160	182.5 ± 58.9	207.2 ± 59.4	0.013
	Total Erros *n* = 160	51.9 ± 36.3	67.1 ± 32.4	0.009
	Total Time *n* = 159	261.9 ± 76.2	283.0 ± 101.0	0.14
VF	Animals *n* = 170	13.4 ± 4.5	9.1 ± 3.6	< 0.001
	Objects *n* = 170	18.2 ± 6.8	11.7 ± 4.4	< 0.001
	Alternated *n* = 170	11.8 ± 4.3	8.1 ± 3.2	< 0.001
	Total *n* = 170	43.4 ± 13.9	28.8 ± 10.1	< 0.001
MoCA	1, *n* = 170	2.43 ± 1.74	1.39 ± 1.37	< 0.001
	2, *n* = 170	2.69 ± 0.86	2.29 ± 1.17	0.011
	3, *n* = 170	4.14 ± 1.68	2.92 ± 1.68	< 0.001
	4, *n* = 142	2.00 ± 1.03	1.54 ± 0.94	0.009
	5, *n* = 169	1.17 ± 0.86	0.68 ± 0.85	< 0.001
	6, *n* = 170	2.13 ± 1.56	1.70 ± 1.34	0.067
	7, *n* = 170	5.85 ± 0.41	5.30 ± 0.76	< 0.001
	MoCA *n* = 170	21.2 ± 4.5	16.3 ± 4.0	< 0.001
	Adjusted MoCA *n* = 170	21.1 ± 4.7	15.7 ± 4.2	< 0.001

## Discussion

This study used 7 instruments to assess the cognitive functions of elder population in a Chinese rural community. The results showed that although almost all the tests were completed, a significant percentage of the tests appeared to not contain valid data. Of all the instrument used, MMSE had the most valid data, also the CI prevalence identified with adjusted MMSE score was within a reasonable range. In contrast, MoCA seemed to acquire less effective data and an abnormally high percentage of CI percentage, although it showed correlation with all the scores acquired with other instruments, indicating a more complete domain overlapping.

MMSE is a 10-min bedside screen tools that consists of 11 questions assessing a variety of domains. It is the most commonly administered brief screen for cognitive functioning. As previously reported, MMSE usually had more accuracy compared to other tools, however still with limitations. For example, MMSE had the highest accuracy when assessing MCI but still could not differentiate MCI from subjective cognitive decline (SCD) [44]. In addition, systematic review identified numerous studies using MMSE as assessment tool for dementia in elderly but a summary diagnostic accuracy could not be estimated due to various cut points and dementia types [45]. MMSE was considered to have good concurrent validity with other neuropsychological assessment instruments but is not highly specific. It is also suggested that MMSE lacks of sensitivity to deficits in domains such as psychomotor speed, problem solving, or attention. It may also be not efficient in detecting early signs of CI. The standard cutoff score for normal cognitive functioning in MMSE is typically at 24 of 30. However, age is associated with a decline in scores, as well as lower education level. This can potentially result in misidentification of CI. For example, in participants 60 to 69 years old who had 0 to 4 years of education, a score of 18 to 19 may be a more appropriate cut score, whereas in the same aged people with ≥13 years of education, a cut score of 26 to 27 is more appropriate [46]. In this study, the MMSE score was adjusted to age and education, and a cutoff of 24 appeared to be applicable. In addition, the MMSE tests acquired the most “valid” results. We reasoned that short time of survey and the insensitivity of MMSE to attention may have resulted in less variation of the results.

Although MMSE is still one of the most frequently used cognitive screening tests, MoCA has gained increased popularity in recent years. It is suggested that the MoCA has a higher sensitivity and similar specificity compared to the MMSE for diseases affecting cognition and may be a better screening method in patients with certain diseases [10]. The original validation study of the MoCA suggested a cut-off value of ≥26 as identification of mild cognitive impairment (MCI). However, it has been recognized that lower thresholds may be necessary due to influences of age and education [47]. In the current study, after adjusting to age and education, with a cutoff of 26, an abnormally high percentage of study population was identified as with CI. Even if we lower the adjusted cutoff to 21, there would still be 59% of CI identified. With the seemingly extreme low score of MoCA, we suspected that the participants may have lost attention during the test process, resulting in a general low score.

Many neuropsychological instruments developed may be used for quick screening of cognitive functions. Verbal fluency test mostly evaluates attention and executive function [43, 48]. Performance on the SDMT is mostly underpinned by attention, perceptual speed, motor speed, and visual scanning [49]. SDMT check the reading speed, verbal operational memory and execution functions, and is particularly sensitive to a reduced speed of information processing [28]. TMT is mostly used to assess executive function by measuring timed motor and visual tasks [36]. DS are commonly used to test memory storage capabilities and numerical skills associated with the functioning of operational verbal memory [50]. All these tests have been utilized to assess cognitive/neuropsychological functions in Chinese population [51, 52]. However, there is rarely systematic analysis of the consistency among these tests. In this study, we found that MoCA scores had significant correlation with all the other instrument scores, suggesting that these instruments had overlapping cognitive domains tested and MoCA may be the most completely designed screening tools. In contrast, MMSE only correlated with MoCA and VF. These results appeared to be consistent with that MMSE is insensitive to attention, but all the other tests are somehow based on the participants’ attention. Since most of our surveys were performed in a public space, the lack of attention, combined with other distractions, may be the main reason that only MMSE had the valid data in almost all the participants.

The strength of the current study is the concentration on Chinese population in which rarely an alike study was available. This was the first systematical assessment of different cognitive function tests in Chinese population with a significant number of participants. The results should provide valuable reference to future epidemiological studies of CI in the country. However, the study is with certain limitations. The sample size was relatively small to confirm the validity of a screen instrument. And the lack of “valid” data mostly caused by our study settings therefore in no way indicating the invalidity of the instrument used. It rather raised a question that the site of performing may be take into consideration when using a cognitive screening instrument. In addition, due to the inconsistent results, we may not draw a definite conclusion on the prevalence of CI in the surveyed participants. Nonetheless, from the perspective of testing instruments in a public survey environment, the results had certain interesting indications.

## Conclusion

In conclusion, using seven cognitive screening instruments in a cross-sectional survey study, we found that MMSE may still present the most applicable tools for quick screen of cognitive functions, especially when environmental conditions may interfere with participants’ attention.

## Data Availability

The datasets used and/or analyzed during the current study are available from the corresponding author on reasonable request.

## Abbreviations

CI: Cognitive impairment
MMSE: Mini-Mental State Examination
MoCA: Montreal Cognitive Assessment
SDMT: Symbol Digits Modalities Test
SCWT: Stroop Color and Word Test
DS: Digit Spans Test
TMT: Trail-making test
VF: Verbal Fluency
ED: Emergency department
MCI: Mild cognitive impairment
